# Co-occurrence of human papillomavirus (HPV) in newborns and their parents

**DOI:** 10.1186/s12879-019-4503-4

**Published:** 2019-11-04

**Authors:** Mariusz Skoczyński, Anna Goździcka-Józefiak, Anna Kwaśniewska

**Affiliations:** 1 Department of Obstetrics and Pathology of Pregnancy, Medical University of Lublin, Lublin, Poland; 2 Department of Molecular Virology, Institute of Experimental Biology, Adam Mickiewicz University, Poznan, Poland

**Keywords:** Maternal infection, Paternal infection, Periconceptional infection, Perinatal transmission

## Abstract

**Background:**

The potential HPV transmission route includes horizontal transmission “in utero” and vertical transmission from parents. Less is known about the role of child’s father as a potential source of HPV infection and involved in the pathogen’s epidemic chain. A possible consequence of perinatal infection includes HPV-related childhood diseases and carrying the risk of cervical cancer development in female offspring. In view of the evidence, studies of HPV co-occurrence in one or both parents and their offspring seem vital for the implementation of respective preventive measures. Consequently, the aim of this study was to determine the incidence of common HPV 16/18 infections in newborns and their parents, and to assess its role of the periconceptional transmission.

**Methods:**

To determine the incidence of common HPV infections in newborns from single pregnancies and their parents. The study included 146 pregnant women, as well as their partners, and newborns. They were tested for the presence of HPV 16/18 DNA using the PCR method. HPV types 16 and/or 18 were identified using type-specific PCR primers. The quality of the extracted DNA was evaluated by PCR using PC03/PC04 β-globin-specific primers. The relationship between the presence of neonatal and parental HPV infection was analyzed using a multivariable regression model. Calculations were carried out with the Statistica 10.

**Results:**

The presence of HPV DNA was detected in 19 (13,01%) newborns, 28 (19,18%) mothers, and 20 (13,7%) fathers. The viral DNA was detected in 14 newborns delivered by HPV-positive mothers (OR = 26,08; CI: 8,07-84,31, *p* < 0.001), 12 descendants of HPV-positive fathers (OR = 22,13; CI: 6,97-70,27, *p* < 0.001), and 10 children originating from two infected parents (OR = 24,20; CI: 6,84–85,57 *p* < 0.001). Those findings points to a increase risk of an acquired infection in newborns with HPV-positive parents.

**Conclusion:**

Our findings suggest the possible role of the periconceptional transmission in the mode of acquired HPV 16/18 infections.

## Background

The potential HPV transmission route includes horizontal transmission “in utero” and vertical transmission from parents. Data presented in various researches show the possibility of perinatal or intrauterine mode of infection [1–4]. Less is known about the role of child’s father as a potential source of HPV infection, although it was revealed that men are involved in the pathogen’s epidemic chain [4–6]. Several authors confirmed the presence of HPV in semen, which raises the possibility of periconceptional infection [6, 7]. Consequently, this route of transmission is postulated to be a potential reason of a persistent viral infection of the child [6, 7]. A possible consequence of perinatal infection includes HPV-related childhood diseases and carrying the risk of cervical cancer development in female offspring [4, 8, 9]. In view of the aforementioned evidence, studies of HPV co-occurrence in one or both parents and their offspring seem vital in order to understand the pathogenesis of pregnancy-related infections with this pathogen as well as for the implementation of respective preventive measures. Consequently, the aim of this study was to determine the incidence of common HPV types 16 and/or 18 infections in newborns and their parents, and to assess the role of the periconceptional transmission.

## Methods

### Patients

This research was conducted between March 2009 and July 2011. The studied group comprised pregnant women who delivered at the Clinic of Obstetrics and Pathology of Pregnancy, Medical University of Lublin (Poland), and their partners. For the study we recruited 146 consecutive parental couples. The gestational age varied from 33 to 41 weeks. Individuals selected for this study met specific inclusion criteria. Among those criteria were: 1) singleton pregnancy, 2) no HPV infection symptoms present in any of the parents, and 3) physiological cervical smear in pregnant woman. Among the exclusion criteria were: 1) previous HPV infections, 2) abnormal cervical smear in pregnant woman, and 3) multiple pregnancy.

The protocol of this study was performed in accordance with the Declaration of Helsinki and approved by Local Bioethical Committee at the Medical University of Lublin (No. KE-0254/143/2007). Before participation in any of the procedures the studied group expressed their consent by completing a written form.

### Samples

Posterior vaginal fornix smear samples as well as peripheral blood samples were collected from the women in the onset of labour. Additionally, buccal mucosal smears were drawn from both parents and their children. Discharge gathered in newborns upper airways was extracted in order for buccal smears to be collected directly after birth. A suitably trained member of the personnel managed the collection of all material. Subsequently the samples were transferred to sterile tubes awaiting for HPV DNA testing (Eurotubo®; Deltalab, Spain). The material was then subject to freezing at − 70 °C pending additional analyses.

### PCR identification

PCR testing was utilized as means of detecting HPV DNA in the collected samples. HPV types distinction was made possible by using PCR primers for L1: MY09: 5′-CGTCCMARRGGAWACTGATC-3′ and MY11: 5′-GCMCAAGGWCATAAYAATGG-3′, where M = A + C, R = A + G, W = A + T, Y=C + T. This set of primers amplifies DNA from at least 33 different HPV genotypes. HPV types 16 and/or 18 were identified using the following type-specific PCR primers: HPV16/L1A/HPV16/L1B, 5′-GCCTGTGTAGGTGTTGAGGT-3′ and 5′-TGGATTTACTCCAACATTGG-3′ product size: 264 bp; HPV18/L1A/ HPV18/L1B, 5-’ GTGGACCAGCAAATACAGGA-3’and 5′- TGCAACGACCACGTGTTGGA-3′, product size: 162 bp; HPV18ME12/HPV18ME50/E6, 5′-CACGGCGACCCTACAAGCTACCTG-3′, and 5′-TGCAGCACGAATTGGCACTGGCCTC-3′, product size: 404 bp. The quality of the extracted DNA was evaluated by PCR using PC03/PC04 β-globin-specific primers. The reaction described above was developed with reference according to Tucker et al. [10]. The total volume of 10 μl of PCR mixture contained 1 μM of primers, 200 μM of deoxynucleotide triphosphates, 1 x PCR buffer (0.1 M Tris-HCl pH 8.8, 0.5 M KCl, 0.015 M MgCl_2_, 1% Triton X-100), the investigated DNA (10 ng/μl), and Tag polymerase at a final concentration of 40 U/ml. After preliminary denaturation (15 min at 94 °C), samples were amplified for 31 cycles in a thermal cycler, consisted of a 30 s period of denaturation at 94 °C, annealing at 59 °C for the same amount of time, followed by primer extension at 72 °C for 1 minute. In the last PCR cycle, the stage of complementary DNA synthesis at 72 °C was extended to 420 s. Agarose gel electrophoresis in the presence of pBluescript DNA digested with HindI was utilized to analyze PCR products. A further HPV DNA detection, and HPV genotypes identification by direct sequencing from PCR reaction tube (without purification from MY09 and MY11 oligonucleotides was performed). The obtained results were subsequently analyzed with BLAST database (http://blast.ncbi.nlm.nih.gov).

### Statistical methods

To verify the normal distribution of continuous variables we use the Kolmogorov-Smirnoff test. Statistical characteristics of these variables were presented as medians and ranges, and their intergroup comparisons were performed using the Mann-Whitney U-test. The distributions of qualitative variables were compared amongst studied groups with the Pearson’s chi-square test and Fischer’s exact test. We use a multivariable regression model to analyze the relationship between the presence of neonatal and parental HPV infection; odds ratios (ORs) were determined. All calculations were carried out with Statistica 10 (StatSoft®, Tulsa OK, USA) package, with the level of significance set at ^*^p ≤ 0.05.

## Results

The examined group consisted of women ages 17 to 43 with a median age of 29. Twenty-eight (19.18%) cases of HPV 16/18 infection were detected amongst the pregnant women participating in this study. In 10 cases, HPV DNA was detected solely in vaginal smears, and in 5 cases solely in the buccal smears; 13 women tested positive for both samples. In two cases, HPV DNA was also detected in the peripheral blood of the pregnant women. There was no significant difference between participants with positive and negative results on HPV DNA testing as regards their demographical and obstetrical features (Table 1).

**Table 1:** Demographic and obstetrical characteristics of HPV types16 and/or 18-positive, and HPV types 16 and/or 18-negative pregnant women

Parameter	HPV16 /18 (+) *n* = 28 (19.18%)	HPV16 /18 (−) *n* = 118 (80.82%)	*p* value
Age (years)	28.5 ± 4.7	29.38 ± 4.59	0.992
Marriage	23 (82.1%)	104 (88.1%)	0.397
Unmarried partnership	5 (17.86%)	14 (11.86%)	0.397
< 10 years of education	2 (7.14%)	4 (3.39%)	0.375
Cigarette smoking	2 (7.14%)	7 (5.93%)	0.812
Age of sexual initiation (years)	19.49 ± 2.77	19.65 ± 2.78	0.785
At least three sexual partners	8 (28.57%)	32 (27.12%)	0.873
Contraceptive use	13 (46.43%)	66 (55.93%)	0.365
Number of pregnancies	1.98 ± 1.032 (1–5)	1.94 ± 0.97	0.847

*n* Number of participants

Twenty (13.7%) cases of HPV 16/18 infection were detected amongst fathers participating in this study as confirmed by the examination of buccal smears. The offspring of study participants included 80 female and 66 male newborns. Neonatal birth weight ranged from 1740 g. to 4670 g. with a median weight of 3405 g. In case of 19 (13.01%) newborns HPV 16/18 DNA was found in the collected samples. In 6 cases the infection was confirmed based on a buccal smear test and in 3 patients through examination of the upper respiratory airway discharge; 10 newborns had positive results of both samples. In one case, HPV was also detected in the umbilical blood sample. Newborns positive and those negative for HPV 16/18 DNA did not differ significantly in terms of their obstetrical characteristics (Table 2).

**Table 2:** Obstetrical characteristics of HPV types16 and/or 18-positive and HPV types16 and/or 18-negative neonates

Parameter	HPV 16/18(+) *n* = 19 (13.01%)	HPV 16/18(−) *n* = 127 (86.99%)	*p* value
Obstetrical characteristics			
Gestational age (weeks)	38.90 ± 2.08	38.12 ± 1.69	0.609
Cesarean section	9 (47.37%)	79 (62.2%)	0.219
Vaginal delivery	10 (52.63%)	48 (37.8%)	0.219
Rupture of membranes > 2 h	6 (31.58%)	25 (19.69%)	0.237
Rupture of membranes ≤2 h	13 (68.42%)	102 (80.31%)	0.237
Neonatal characteristics			
Female gender	11 (57.89%)	69 (54.33&)	0.769
Male gender	8 (42.11%)	58 (45.67%)	0.769
Birth weight (g)	3261.03 ± 562.94	3413.12 ± 575.05	0.283

*n* Number of participants

In the case of 15 of the 28 HPV 16/18-positive women, the virus’ genetic material was also isolated in their spouses. The presence of HPV 16/18 DNA was detected in 14 of the 28 newborns delivered by mothers in whom an HPV infection was confirmed based on the examination of at least one biological sample, in 12 of the 20 descendants of HPV 16/18 -positive fathers, and in 10 of the 15 children originating from two infected parents. The following variables (in descending order of risk) proved to be significant risk factors for the acquiring of a neonatal HPV infection on the multivariate analysis of logistic regression: 1) maternal infection confirmed by a positive result of at least one examined sample (OR = 26.08; CI:8.07–84.31, *p* < 0.001), 2) infection of both parents (OR = 24.2; CI:6.84–85.57, *p* < 0.001), 3) maternal infection confirmed by a positive vaginal smear test (OR = 21.54; CI:6.94–66.87, *p* < 0.001), and 4) maternal infection confirmed by a positive buccal smear test (OR = 10.64; CI:3.47–32.63, *p* < 0.001). In contrast, the risk of neonatal infection was not modulated by the detection of HPV DNA in maternal blood (OR = 6.58; CI:0.39–112.31, *p* = 0.191) and by the number of HPV-positive maternal samples (OR = 0.98; CI:0.21–4.68, *p* = 0.979) (Table 3).

**Table 3:** Odds ratio (OR) of detecting HPV 16/18 genetic material in studied neonates depending on the characteristics of HPV 16/18 infection in their parents

Variable	OR	95%CI	*p* value
Maternal infection – any sample	26.08	8.07–84.31	0.000
Infection of both parents	24.20	6.84–85.57	0.000
Paternal infection	22.13	6.97–70.27	0.000
Maternal infection – vaginal smear	21.54	6.94–66.87	0.000
Maternal infection – buccal smear	10.64	3.47–32.63	0.000
Maternal infection – peripheral blood	6.58	0.39–112.31	0.191
Number of positive maternal samples	0.98	0.21–4.68	0.979

## Discussion

Recently, the possibility of perinatal transmission of HPV infection has been postulated [1, 2, 11]. In this study, we confirmed the presence of HPV 16/18 DNA in nearly 20% of examined women. Rintala M.A. and Brun-Micaleff E. reported closely resembling results in terms of prevalence of HPV infection in pregnant women [12, 13]. Nonetheless the prevalence rate of HPV detection may vary from 5,5% up to 65%, as shown by a metanalysis of 9 different studies conducted on a collective number of 2111 of pregnant women [1].

These differences can result from several causes. The main being: inhomogeneous selection standards of the study material, a diversity of methods for HPV DNA detection and relatively small quantity of the study samples [14]. False negative results may be caused by insufficient number of viral copies in the studied samples [15]. Another reason of false positive results could be a secondary contamination of the collected samples. Irrespective of this, the most frequent type of HPV isolated in pregnant women and their offspring is the highly oncogenic HPV 16 [9, 16].

Our study revealed a 13% prevalence of neonatal HPV 16/18 infection, like to that observed in Bandyopadhyaya’s et al. - a study including a similar amount of pregnant women and comparable techniques as in our group [17]. It has been postulated that the offspring of HPV infected mother have an increased risk of infection [9, 12, 18]. The reported frequency of HPV infection acquired during perinatal period is still highly ambiguous. Other sources claim that HPV DNA is isolated in between 1 to 18% of newborns delivered by HPV-positive mothers without clinical signs of infection [2, 19, 20]. Women with clinical symptoms of the infection bear higher risk of their children being infected i.e. between 4 and 79% [21]. The presence of HPV types 16 and/or 18 in our study group was detected in 14 out of 28 newborns delivered by HPV 16/18- positive mothers without clinical symptoms of infection. This finding points to a significant risk of an acquired infection in newborns with HPV-positive mothers. Moreover, the statistical results of logistic regression (OR = 26.08), also support this fact. Furthermore, our study revealed the presence of HPV types 16 and/or 18 genetic material in 13.7% of paternal buccal smears. In as many as 12 out of 20 cases, a paternal infection corresponded to the presence of HPV 16/18 DNA in newborns. We have found only a few published reports on the co-occurrence of HPV infection in men and their offspring [7, 22, 23]. Rintala et al. revealed that a parental HPV infection is associated with a 11% probability of an acquired neonatal infection [12]. The marked difference between this value and the respective rate observed in our study could result from the aforementioned differences in the research methodology. Nonetheless, the high risk of acquired infection in the offspring of HPV-positive fathers (OR = 22.13) documented in this study substantiates further research in this matter. Our findings suggest that the presence of HPV genetic material in the father modulates the risk of neonatal infection to a similar extent as a maternal infection; it seems obvious when the sexual route of Human Papillomavirus transmission is taken into consideration. The transmission of HPV from previous intimate partners as well as the presence of an acquired infection in the mother could explain slightly lower odds ratio values of a paternal infection as compared to ORs of a maternal infection.

In more than half of the HPV-positive cases (15/28), an infection in the mother co-existed with that of the father, and in nearly 70% of cases the infection of both parents corresponded to the presence of HPV genetic material in neonatal tissues. These findings and that of the logistic regression analysis (OR = 24.20) point to the possible role of the periconceptional transmission of the pathogen in the pathogenesis of acquired HPV infections. This substantiates further research in this direction, as well as the consideration of HPV testing of both future parents in the protocol of preconception care.

Aside from this important conclusion, our findings suggest that testing vaginal or buccal smears in pregnant women (OR = 21.54 and OR = 10.64, respectively; *p* = 0.000 in either case) can be more useful for the risk assessment of intrauterine HPV transmission than the virology examination of peripheral blood (OR = 6.58, *p* = 0.191). This finding seems particularly attractive in the context of the popularization of non-invasive methods of virology diagnosis. The identification of parents in whose swabs of the oral mucosa HPV DNA was detected can help emerge a group of children who run a relatively significant risk of being infected.

## Conclusion

Our findings suggest the possible role of the periconceptional transmission in the mode of acquired HPV 16/18 infections.

## Data Availability

The datasets used and/or analyzed during the current study are available from the corresponding author on reasonable request. Main data generated or analyzed during this study are included in this published article [and its supplementary information files].

## Abbreviations

HPV: Human Papillomavirus
OR: Odds ratio
PCR: Polymerase Chain Reaction
